# DATABASE, *The Journal of Biological Databases and Curation*, is now the official journal of the International Society for Biocuration

**DOI:** 10.1093/database/bat077

**Published:** 2013-12-06

**Authors:** Pascale Gaudet, Monica Munoz-Torres, Marc Robinson-Rechavi, Teresa Attwood, Alex Bateman, J. Michael Cherry, Renate Kania, Claire O'Donovan, Chisato Yamasaki

**Affiliations:** ^1^Swiss Institute of Bioinformatics, Geneva, Switzerland, ^2^Lawrence Berkeley National Laboratory, USA, ^3^University of Lausanne, Switzerland, ^4^Faculty of Life Sciences and School of Computer Science, The University of Manchester, UK, ^5^European Bioinformatics Institute, Wellcome Trust Genome Campus, Hinxton, UK, ^6^Department of Genetics, Stanford University, Stanford, CA, USA, ^7^Heidelberg Institute for Theoretical Studies, Heidelberg, Germany and ^8^National Institute of Biomedical Innovation (NIBIO), Osaka, Japan

The International Society for Biocuration (ISB) was created in 2009 specifically to promote biocuration, the product of multidisciplinary teams of database curators, software developers and bioinformaticians. Biocurators, whose work facilitates research and education across the life sciences, create and maintain a wide variety of online tools and databases, covering topics as diverse as biochemical structures, chromosomal features and phenotypes of mutant genes. These resources are now so essential to the biological community that databases have become integral to the daily work of most researchers. But biological databases are far from static: in addition to the need to constantly capture new knowledge (from the literature, from other databases, from analysis tools, etc.), data representation must also keep pace with current research—new data types must be modeled—and improved methods of data storage, representation and analysis must be continually developed. Nevertheless, such important efforts have not always been recognized, and have often not been published in full, owing to the lack of a suitable journal.

DATABASE, *The Journal of Biological Databases and Curation*, was launched in 2010 to support the growing need of the research community to discuss a range of issues related to the creation, development and maintenance of biological databases, and to strengthen communication between database developers, curators and users.

As this resonates strongly with the mission of the ISB, we are delighted to announce that DATABASE has now become the Society’s official journal. The scope of DATABASE includes many areas relevant to the endeavors of the biocuration community. Moreover, DATABASE is an open-access journal, which is critical for biocuration worldwide and one of the core values that the ISB promotes. Since its creation, DATABASE has published more than 250 articles, 50 of which have appeared in the *Biocuration Virtual Issue*, a special collection of articles describing work presented at the annual International Biocuration Conference.

Scholarly exchanges among scientists are invaluable for helping a discipline to realize its full potential. To this end, DATABASE and the ISB are excited to be able to work together more closely, a collaboration that we expect will enhance the visibility and impact of biocurators’ work, and hence to increase the value of the Journal to members both of the ISB and of the wider scientific community.

## The Executive Committee of the International Society for Biocuration

Pascale Gaudet (Chair), Monica Munoz-Torres (Secretary), Marc Robinson-Rechavi (Treasurer), Teresa Attwood, Alex J. Michael Cherry, Renate Kania, Claire O'Donovan and Chisato Yamasaki.

